# Urokinase-type Plasminogen Activator Induces Neurorepair in the Ischemic Brain

**DOI:** 10.17756/jnen.2018-039

**Published:** 2018-09-26

**Authors:** Paola Merino, Manuel Yepes

**Affiliations:** 1 Division of Neuropharmacology and Neurologic Diseases, Yerkes National Primate Research Center; Atlanta, GA, USA; 2 Department of Neurology, Emory University School of Medicine; Atlanta, GA, USA; 3 Department of Neurology, Veterans Affairs Medical Center; Atlanta, GA, USA

**Keywords:** Urokinase-type plasminogen activator (uPA), Urokinase-type plasminogen activator receptor (uPAR), Neurorepair, Ischemic brain

## Abstract

Urokinase-type plasminogen activator (uPA) is a serine proteinase that upon binding to its receptor (uPAR) catalyzes the conversion of plasminogen into plasmin on the cell surface. Recent studies indicate that neurons but not astrocytes release uPA during the recovery phase from an ischemic injury, and that binding of uPA to uPAR promotes neurorepair in the ischemic brain by a mechanism that does not require plasmin generation. A combined approach of *in vitro* and *in vivo* studies has shown that uPA binding to uPAR induces the reorganization of the actin cytoskeleton in dendritic spines and axons that have suffered an ischemic injury. Furthermore, recent data indicate that uPA-uPAR binding induces astrocytic activation and a crosstalk between activated astrocytes and the injured neuron that triggers a sequence of biochemical events that promote the repair of synapses injured by the ischemic insult. The translational relevance of these observations is noteworthy because following its intravenous administrations recombinant uPA (ruPA) reaches the ischemic tissue, thus raising the question of whether treatment with ruPA is an effective therapeutic strategy to promote neurorepair functional recovery among ischemic stroke survivors.

## Introduction

The last two decades have witnessed significant advances in the treatment of acute ischemic stroke patients. Indeed, thrombolysis with recombinant tissue-type plasminogen activator (rtPA) [1], the removal of the occluding clot with different intravascular devices [2], and the implementation of intensive care units and exclusive dedication of vascular neurologists to the diagnosis and treatment of acute ischemic stroke patients [3], have resulted in an almost 19% reduction in stroke-related mortality [4]. However, this decrease in mortality has led to a sharp increase in the number of ischemic stroke patients that survive with different degrees of disability. Hence, the National Stroke Association estimates that while 35% of stroke survivors recover completely or only with minor impairments, 40% to 60% experience severe disability, and 10% require continuous care in a nursing home or other long-term care facilities. Unfortunately, to this date we have no therapeutic strategy to promote functional recovery among these patients. Below we will review recent advances indicating that the plasminogen activation (PA) system, more specifically uPA, plays a pivotal role in the process of neurorepair following an ischemic injury, and how uPA and its receptor (uPAR) that can be used as targets for the design of an effective strategy to promote neurorepair in the ischemic brain. With that in mind, first we will review some basic concepts of neurorepair after an ischemic stroke. Then we will introduce the PA system and its importance for neurorepair after an ischemic injury, to conclude with a discussion of how this knowledge can be translated into the design of therapeutic tools to promote functional improvement following an ischemic stroke.

## Neurorepair in the Ischemic Brain

Due to their high metabolic rate and an almost complete dependence on glucose as energy substrate, neurons are highly vulnerable to cerebral ischemia. Indeed, while 20–40 minutes of interruption of the blood flow to the heart are needed to kill a cardiac myocyte, only 5 minutes of cerebral ischemia are sufficient to induce the death of vulnerable neurons [5]. These observations led us to propose the concept of neuroprotection as the cornerstone for the recovery of neurological function following an ischemic stroke. However, it was soon evident that cerebral ischemia has a rapid and deleterious effect on synaptic structure and function [6] that is not always associated with neuronal death [7], and that synaptic loss and regeneration underlie the functional impairment and recovery in acute ischemic stroke patients [8]. In other words, synaptic protection in surviving neurons should be considered as the first therapeutic target to prevent neurological impairment and promote neurorepair in the ischemic brain. Axonal loss is another cause of neurological impairment following an acute ischemic injury [7], and is estimated that axonal damage is responsible for the long term disability observed in 32% of ischemic stroke survivors. Unfortunately, axonal repair in the ischemic brain is precluded by the development of a microenvironment that inhibits the recovery of axons injured by the ischemic injury [9]. Indeed, the regeneration of injured axons *in vivo* is impaired by the production in the ischemic area of several glial-derived inhibitors such as myelin-associated glycoprotein (MAG), the reticulon protein family member NogoA, oligodendrocyte myelin glycoprotein (OMgp), and chondroitin sulfate proteoglycans [10]. Importantly, although these inhibitors seem to play a central role in brain development and their antagonism has been associated with several neuropsychiatric disorders [11], they prevent axonal repair following an ischemic injury. Strikingly, the data discussed below indicate that uPA/uPAR binding triggers the activation of cell signaling pathways that are able to overcome the growth inhibitory effect generated in the ischemic brain by these proteins. These findings agree with the concept that a successful therapeutic strategy to promote axonal recovery not only should activate the biochemical machinery that promotes axonal repair, but also overcome this inhibitory microenvironment that prevents axonal recovery.

A substantial body of experimental evidence indicates that neurons situated in a thin band of brain tissue surrounding the necrotic core survive the ischemic injury despite exhibiting signs of axonal degeneration and loss of dendritic spines [12]. Importantly, it has been shown that these neurons are capable of activating cell signaling pathways associated with neuronal survival, such as those activated by vascular endothelial growth factor (VEGF) and brain-derived neurotrophic factor (BDNF) [13], and that these events drive the process of neurorepair in the ischemic brain. Indeed, BDNF binding to TrkB receptors triggers the phosphorylation of a tyrosine residue in its cytoplasmic domain which activates several cell signaling pathways, among which the phosphatidylinositol-3 kinase has reported to have a protective effect in the ischemic brain [14]. Furthermore, several studies indicate that besides its neuroprotective effect, BDNF also may induce neurorepair and improved neurological outcome following an acute ischemic injury [15]. Importantly, plasmin activates BDNF by inducing the cleavage of pro-BDNF into mature BDNF [16]. These observations are of particular importance, because we have found that the PA system is activated in the periinfarct region where it promotes axonal and dendritic repair in the brain that has suffered an ischemic injury.

## The Plasminogen Activation System

The PA system is an enzymatic cascade of proteases and their inhibitors that was initially thought to be solely involved in the conversion of plasminogen into plasmin by the activity of two serine proteases: tissue-type plasminogen activator (tPA) and uPA [17]. However, soon thereafter it was found that fibrinolysis is not the only role of tPA and uPA, and instead that both PAs are also found in the synapse where they play a central role in the development of synaptic plasticity via mechanisms that not always require plasmin generation [18–23]. Significantly, cerebral ischemia induces the secretion of both proteases into the synaptic cleft [24, 25], but in contrast with tPA that is rapidly released after the onset of the ischemic injury, uPA is secreted only during the recovery phase [25]. Curiously, and in sharp contrast with tPA, the role of uPA in the ischemic brain remained largely unknown until few years ago. Furthermore, although some investigators considered uPA to be a redundant system with no functional implications in brain function *in vivo*, today is clear that uPA and tPA play distinct roles in the ischemic brain with a significant impact on neuronal survival and functional recovery. Below we will discuss what is known to this date on the role of uPA in the ischemic brain.

## Urokinase-type Plasminogen Activator (uPA)

uPA is assembled by an amino-terminal domain that harbors the binding site to its receptor (uPAR), a kringle domain with a sequence that interacts with plasminogen activator inhibitor-1 (PAI-1), and a catalytic domain located in the carboxyl terminus that has the protease domain. uPA is secreted as a 54 kD single-chain polypeptide that was originally considered as an inactive enzyme precursor (prourokinase). However, it was soon evident that prourokinase can convert plasminogen into plasmin, which then cleaves the single chain polypeptide at Lys158-Ile159, converting it into a two-chain form with protease activity [26]. Most of the biological effects of uPA are mediated by its binding to uPAR, a glycosylphosphatidylinositol (GPI)-anchored protein with three domains (D1, D2 and D3) with high affinity for single- and two-chain uPA and uPA’s amino terminal factor (ATF; assembled by the first two domains of uPA) [27]. Importantly, uPAR lacks a cytosolic domain, and thus needs transmembrane co-receptors, such as integrins [20, 23, 28] (transmembrane receptors assembled by α and β subunits that bind to different ligands in the extracellular matrix such as laminin, collagen, fibronectin, and vitronectin [29]), and the low density lipoprotein receptor-related protein 1 (LRP1; a member of the low density lipoprotein receptor gene family [20, 30], to activate intracellular signaling pathways via plasminogen-dependent and -independent mechanisms.

## uPA and uPAR in the Ischemic Brain

uPA and uPAR expression in the ischemic brain. The expression of uPA and uPAR in the brain varies significantly across the developmental span. Accordingly, although during the early phases of development all neurons express uPA [31], in the adult brain uPA is found only in well-circumscribed groups of neurons in the hippocampus and some subcortical structures [32, 33]. Likewise, while uPAR is found in all neuronal extension early in development, only growth cones and some dendritic spines and astrocytes express this receptor in the adult brain [20, 22, 25]. Interestingly, we found that during the recovery phase form an ischemic injury the expression of uPA increases again to levels observed during development [25]. Furthermore, immunocytochemical studies indicate that the expression of uPAR increases in dendrites and growth cones, also to levels comparable to those seen during development [20]. It is important to note that this pattern of uPAR expression is also observed in other experimental models of injury with non-neuronal cells [34]. To further characterize these observations, we measured the release of tPA and uPA in the culture medium of cerebral cortical neurons exposed to 55 minutes of oxygen and glucose deprivation (OGD) conditions. We found that while neurons release tPA within minutes of exposure to OGD [24], the concentration of uPA increases only one hour after the end of the hypoxic injury [25]. This delayed increase in uPA and the fact that genetic deficiency of uPA (uPA^−/−^) does not have an effect on OGD- or cerebral ischemia-induced neuronal death *in vivo* and *in vitro* [25] strongly suggested that the release of neuronal uPA may play a role in the process of neurorepair following an ischemic injury.

## uPA Promotes Neurological Repair

A better understanding of the role of uPA and uPAR in the ischemic brain was provided by the observation that compared to wild-type (Wt) littermate controls, animals genetically deficient on either uPA or uPAR (uPAR^−/−^) have incomplete neurological recovery following transient occlusion of the middle cerebral artery (tMCAO). Importantly, intravenous administration of recombinant uPA (ruPA) after tMCAO reverted the phenotype of uPA^−/−^ but not uPAR^−/−^ animals, indicating that uPA promotes functional improvement following an ischemic stroke, and that this effect is mediated by its binding to uPAR [25].

UPA/uPAR binding promotes dendritic spine recovery. Because changes in fractional anisotropy (Fa) and mean diffusivity (MD) of water measured with diffusion tensor imaging (DTI) provide reliable information on the microneuroanatomy of the brain [35], we then measured the Fa and MD of water in the brain of Wt and uPA^−/−^ mice 24 hours after tMCAO. We found a significant decrease in Fa and an increase in MD of water in a thin band surrounding the necrotic core of Wt but not uPA^−/−^ animals. Importantly, confocal microscopy studies indicated that the number of axonal projections and the magnitude of cerebral edema was comparable between both strains of mice, and thus that these findings could not be accounted by changes in the permeability of the blood-brain barrier (BBB) or differences in the number of neuronal extensions. However, further observations with 3D confocal microscopy revealed the presence of smooth dendrites in uPA^−/−^ mice in contrast with dendrites of Wt animals that harbor a significant number of filopodia and clusters of dendritic spines. Further studies with golgi staining in brain sections of Wt, uPA^−/−^, and uPAR^−/−^ animals indicated that genetic deficiency of either uPA or uPAR prevents the recovery of dendritic spines following an ischemic lesion. Additionally, the use of Plau^GFDhu/GFDhu^ mice in which a 4 aa substitution into the growth factor domain of uPA precludes its binding to uPAR while preserving other functions of the protease and its receptor [36] indicated that binding of endogenous uPA to uPAR in the perinecrotic area promotes the recovery of dendritic spines following an ischemic stroke. These studies were followed by biochemical and immunocytochemical analyses that show that uPA binding to uPAR promotes the reorganization of the actin cytoskeleton in the injured spine via Rac-1-mediated plasminogen-independent induction of profilin expression and cofilin phosphorylation at Ser3 [25]. These data are in line with observation in different experimental systems indicating that uPA/uPAR binding promotes reorganization of the actin cytoskeleton in cells exposed to various experimental conditions [27, 37].

Ezrin, radixin and moesin (ERM) are a group of evolutionarily-conserved proteins that regulate the reorganization of the actin cytoskeleton in different cell systems [38]. A more complete understanding of the effect of uPA/uPAR binding on dendritic spine recovery in the ischemic brain was attained when a combination of proteomic and biochemical studies indicated that uPA induces the expression of ezrin in dendritic spines via a mechanism that does not require plasmin generation. This work revealed that uPA/uPAR binding induces not only the synthesis of ezrin but also its recruitment to the post-synaptic density via β3-integrin and the intercellular adhesion molecule-5 (ICAM-5), and its subsequent β3-integrin-mediated activation by phosphorylation [23]. Additionally, it was demonstrated that uPA-induced synthesis and activation of ezrin promotes the reorganization of the actin cytoskeleton in the injured dendritic spine, and that this leads not only to its recovery but also to the formation of proximal dendritic branches [23]. The finding that uPA has an effect on ICAM-5 is particularly relevant because this intercellular adhesion molecule is pivotal for dendritic outgrowth and the formation and maintenance of dendritic filopodia [39].

uPA induces axonal recovery in the ischemic brain. The observation that in the adult brain uPA is found in few growth cones led us to study whether uPA/uPAR also had an effect on axonal recovery. To answer this question, we performed an immunocytochemical study with anti-uPAR antibodies in axons cultured from cerebral cortical neurons at various time-points after the induction of a mechanical injury. These experiments revealed that uPAR is abundantly expressed in the peripheral region of growth cones re-emerging from the injured site. Subsequent studies showed that treatment with uPA accelerated the formation of new growth cones and the re-emergence of axons from the injured border by a mechanism that did not require plasmin generation but was mediated uPAR- and its co-receptors β1 integrin- and LRP-1-mediated Rac-1 activation [20]. These results were translated into an animal model of white matter ischemia that show that treatment with ruPA promotes axonal recovery and functional improvement via its binding to uPAR following an ischemic lesion to the axons of the internal capsule. Together, these data indicate that uPA binding to uPAR promotes axonal recovery and functional improvement following a mechanical injury *in vitro* and an ischemic lesion *in vivo*.

uPA promotes a cross-talk between astrocytes and the injured synapse. A growing body of experimental evidence indicates that astrocytes play a central role in the recovery process from an ischemic injury [40]. In line with these observations our group found that exposure to OGD induces the recruitment of uPAR to the astrocytic plasma membrane and ERK ½-regulated STAT3-regulated astrocytic activation *in vivo* in an animal model of tMCAO [22]. More importantly, the use of a system of astrocyte-neurons co-cultures indicated that astrocytes activated upon uPA/uPAR binding release thrombosponin-1 (TSP-1), and that the interaction between astrocytic TSP-1 and synaptic LRP1 promotes synaptic recovery in neurons that have suffered a hypoxic injury [22].

## Conclusion and Future Directions

**Figure 1:**
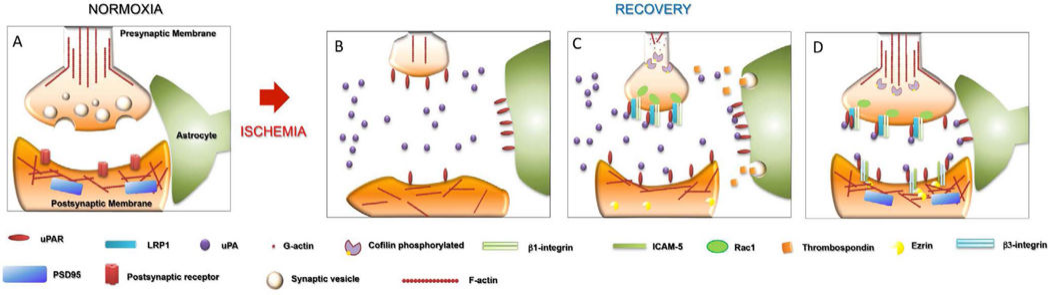
Proposed mechanisms for uPA-induced neurorepair in the ischemic brain. **(A)** Under normoxic conditions the tripartite synapse is assembled by the pre- and postsynaptic terminals encased by perysinaptic glia. **(B)** During the recovery phase from an ischemic injury, neurons release uPA (purple circles) and recruit uPAR (red ovals) to the plasma membrane. **(C)** uPA binding to uPAR activates intercellular signaling pathways specific for the pre- or postsynaptic terminals. So, in the presynaptic terminal uPA/uPAR binding induces Rac-1- , LRP-1 (blue cylinders) and β1 integrin (light green rectangles)-mediated reorganization of the actin cytoskeleton. Furthermore, binding of neuronal uPA to astrocytic uPAR promotes the release of astrocytic thrombosponin 1 (yellow cubes) which promotes synaptic recovery upon interacting with LRP-1 in neurons. **(D)** uPA binding to uPAR on the postsynaptic terminal promotes β3-integrin (light blue rectangles) binding to ICAM-5 (dark green rectangles), which is followed by the recruitment of ezrin (yellow semicircles) to the plasma membrane leading to the reorganization of the actin cytoskeleton in the postsynaptic density.

The data discussed above suggest a model in which the release of uPA by neurons that have suffered an ischemic injury promotes synaptic recovery at multiple levels, by reorganizing the actin cytoskeleton in the pre- and postsynaptic terminals and triggering a crosstalk between astrocytes and the injured synapse that leads to its recovery after the induction of a hypoxic injury (Figure 1). Future studies need to be undertaken to understand the role of plasminogen in this process, and whether it requires plasmin generation. Indeed, the role of plasmin may be cell type- or even cellular compartment-dependent. These studies will be of great translational importance since plasmin generation has a deleterious effect on the permeability of the and this may pose a risk for the potential use of ruPA to promote neurological recovery in ischemic stroke survivors with an already damaged BBB. It is also important to have a better understanding of the signaling processes involved in this neurorestorative effect. Indeed, although now we have good information about the uPAR co-receptors that mediate the restorative effect of uPA in the ischemic brain, we still need to identify the different signaling pathways involved in this process. This information is likely to reveal new targets for the development of an effective therapeutic strategy to promote functional recovery among ischemic stroke survivors.

Studies published to this date indicate that uPA has a neurorestorative effect not only in the cerebral cortex but also in subcortical axons. However, it will be interesting to know if this effect is specific to neurons of the frontoparietal cortex and axons of the internal capsule that have been used in the studies published to this date, or if uPA/uPAR binding also have a neurorestorative effect on other brain structures. Moreover, it needs to be investigated whether the recovery of axons and dendrites induced by uPA results in the formation of new tracts and connections between the area affected by the ischemic injury and the healthy brain. Importantly, if should also be investigated if the synaptic effect of uPA also leads to the formation of aberrant connections and tracts with harmful consequences for brain function. Once these and other questions are answered, preclinical and clinical studies are warranted to determine if treatment with ruPA can be used to improve functional outcome in acute ischemic stroke patients. Indeed, the fact that ruPA can be safely administered to acute ischemic stroke patients [41] greatly facilitates the implementation of potential clinical studies with ischemic stroke survivors. Furthermore, the identification of the domain of uPA that induces neurorepair may further facilitate the design and improve the safety of these potential studies.
